# A cross-sectional survey on fruit bat-human interaction in Pakistan; one health perspective

**DOI:** 10.1186/s42522-023-00078-1

**Published:** 2023-02-28

**Authors:** Touseef Ahmed, Osama Bin Amjad, Haseeb Ahmed, Shafique Ahmed, Jamil Ahmed Ansari, Robert Ricketson, Muhammad Farooq Tahir

**Affiliations:** 1 Department of Epidemiology and Public Health, University of Veterinary and Animal Sciences, Lahore, Pakistan; 2 Department of Biological Sciences, Texas Tech University, Lubbock, TX USA; 3 Department of Meat Sciences and Technology, University of Veterinary and Animal Sciences, Lahore, Pakistan; 4 The Superior University, Lahore, Pakistan; 5 National Institute of Health, Islamabad, Pakistan; 6 Hale O’mana’o Research, Edmond, OK USA; 7 Food and Agriculture Organization (FAO), United Nation, Islamabad, Pakistan

**Keywords:** Human bat interaction, KAP survey, One health, Nipah, Rabies, Megabats

## Abstract

**Objective:**

Several factors, such as residential area topography, population density, and lack of infrastructure, were hypothesized to contribute toward respondents’ knowledge, attitude, and practice regarding disease transmission. The present study was designed to investigate the knowledge, attitudes, and perception of human-fruit bat interaction by student respondents located in ten districts within the Punjab and Khyber Pakhtunkhwa provinces in Pakistan.

**Method:**

A cross-sectional survey was conducted by trained enumerators in academic institutions using a structured questionnaire among student respondents (*n* = 1466), living in two topographically distinct (Mountainous and Plain) residential regions of the Punjab and Khyber Pakhtunkhwa (KPK) provinces in Pakistan regarding their history of bat encounters.

**Results:**

Our study revealed that 71.4% of the 1466 respondents had observed bats in their geographic region. 21% of our survey respondents reported bat bites incidents over their lifetime, but only 40% actively sought medical care for wound management despite reporting they had a close family member that had contracted rabies (27–35%). Our generalized linear models (GLMs) highlighted that a respondent residing in a residential region had a greater association with reporting a suspected bat bite over their lifetime and reported rabies victims in both near and extended family members (OR = −0,85, *p*-value = 0.03, 95% CI). This appeared to be due to delaying consulting a doctor or medical facility for treatment following a suspected bat bite in the topographic residential group as compared to the respondents in the provincial residential group (OR 1.12, *p*-value = 0.04, 95% CI).

**Conclusion:**

Our findings indicate the necessity of a One Health comprehensive surveillance system in Pakistan for emerging and re-emerging zoonotic pathogens in *Pteropodidae*.

## Introduction

Many studies have confirmed a host-parasite relationship between bats and a myriad of viral pathogens. Bat viruses have disrupted global health and economies because of their virulence relative to other mammalian and avian reservoirs [1, 2]. Zoonotic virus diversity within the Indian flying fox (*Pteropus medius,* previously known as *Pteropus giganteus*) population has been previously reported, including highly virulent and pathogenic viruses such as flaviviruses, henipavirus, coronaviruses and hantaviruses [3, 4]. A novel non-RABV lyssavirus, Gannoruwa bat lyssavirus (GBLV), has been recently isolated from Indian flying foxes [5], and another report demonstrated serological evidence of a yet-to-be-identified lyssavirus in fruit bats [6, 7] in India. Phylogenetic analysis of bat lyssaviruses prides some evidence of evolutionary host-shifting events from dogs, other meso-carnivores, and bats [7].

These fruit eating bats belong to the Old-World bat family *Pteropodidae* and are distributed throughout the tropics and subtropics regions of the world. This bat family comprises 196 bat species that feed primarily on 188 plant genera from 64 families [8]. The loss of natural food sources due to clearing of native forest and the subsequent shift by these fruit bats to feeding on agricultural fruit crops can lead to a conflict with humans [8], consequently becoming an additional virus exposure source for human and other wildlife [9, 10]. These ongoing interactions further suggest that these bats can be involved in maintaining different zoonotic diseases cycles through intermediate hosts [11], directly via a bat bite [10, 12], or ingestion of infected bushmeat by humans and wildlife such as mesocarnivores [13] and companion animals [14–17].

Rabies exposures in humans is extremely prevalent across the Indian subcontinent. Half of all human rabies occurs in Asia and there are reported at least 30,000 deaths annually across the Indian Subcontinent. Pakistan alone reports an estimated 6000 rabies related human deaths annually at a rate of 7–10 per million, nearly 10% of the global burden of human rabies [18–20]. In Pakistan, only carnivores, particularly the domestic dog, have been reported as transmitting rabies to humans. No human case of clinical rabies has yet been identified following a bat bite in Pakistan.

Nipah virus outbreaks have occurred sporadically over the past twenty years in the Indian subcontinent. Initially observed as a porcine-human transmission, the natural reservoir for Nipah virus has been identified in the *Pteropus* genus of fruit bat. Since its initial emergence in 1998, there has been reported an alarming human case-fatality rate of 43–75% [21, 22] and is therefore considered to be among the most lethal viruses to humans currently known [23]. Monitoring for Nipah virus and lyssaviruses in fruit bats in Pakistan has yet to be implemented [5, 24]. Human and animal rabies deaths in Pakistan are primarily diagnosed through clinical signs, therefore additional characterization of the lyssavirus and identification of the causative species is rarely conducted [25].

Knowledge, Attitude and Practice (KAP) surveys are used to assess public health engagement, awareness for wildlife education, and conservation efforts [9, 26]. Towards this goal, we report baseline data for planning, application and evaluation of bat associated pathogens management programs [9, 27–29]. This study will lay the groundwork for future studies and policies to involve bats in viral pathogen surveillance and control initiatives [30–33].

## Materials and methods

### Study area

**Fig. 1:**
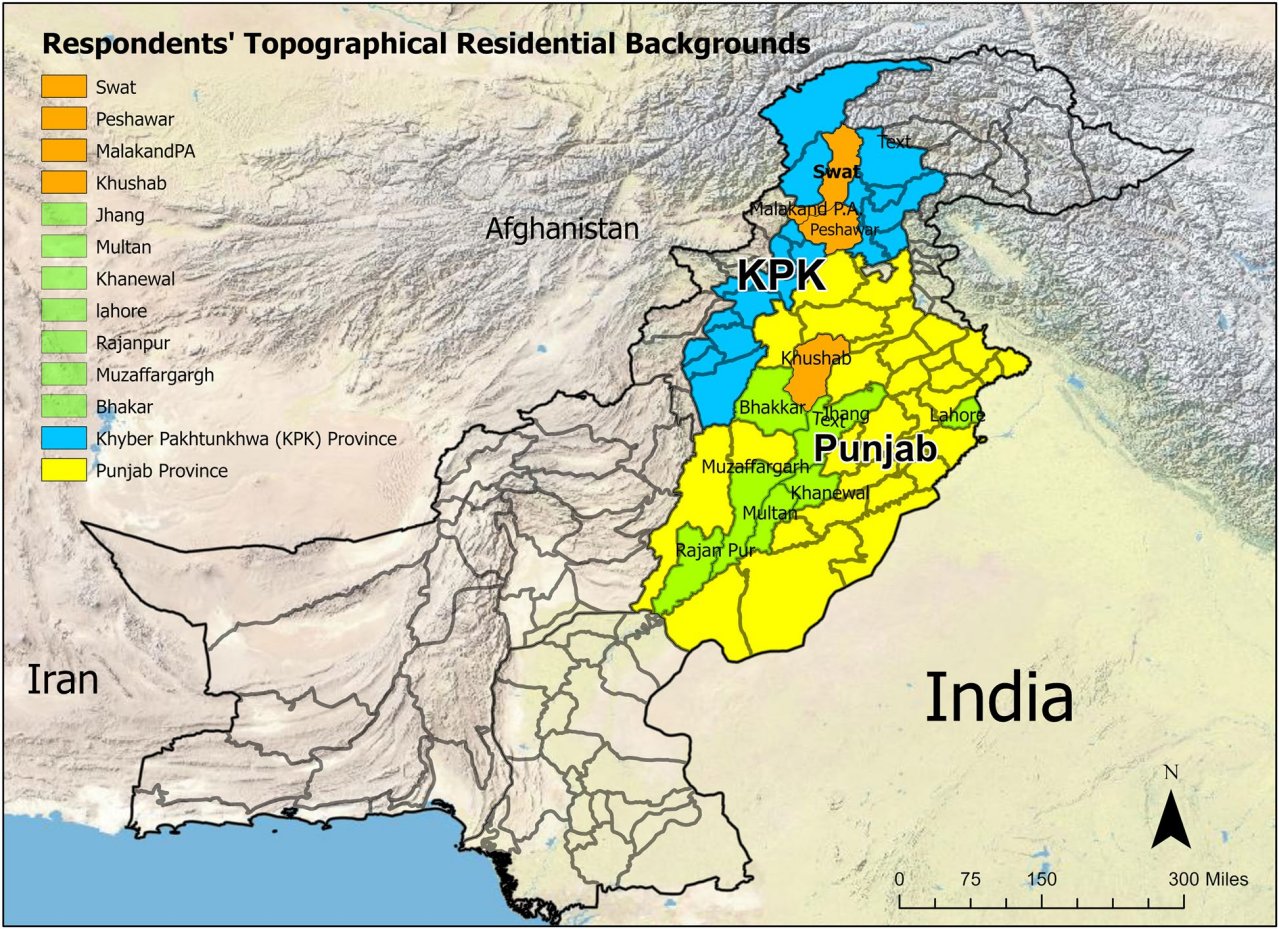
Map of study districts in mountainous and plain topograhical backgrounds in Punjab and Khypber Pakthunkhwa province of Pakistan, A Cross-Sectional Survey on Fruit Bat-Human Interaction in Pakistan; One Health Perspective, 2022

A cross sectional survey was conducted in the Punjab and Khyber Pakhtunkhwa (KPK) provinces in Pakistan. There are two distinct topographies (Mountainous and Plain) within each province. Six districts in the Punjab province were identified as having primarily Plain topography (Lahore, Jhang, Bhakar, Multan, Muzaffargarh, Khanewal). Four districts within the Punjab and Khyber Pakhtunkhwa (KPK) provinces were identified as having a Mountainous topography. Three mountainous districts are in the KPK province (Peshawar, Swat, and Malakand P.A.) and one district is in the Punjab province (Khushab) (Fig. 1).

Face to face surveys were conducted from October 2018 to February 2019 by nine enumerators. The survey enumerators were veterinarians with training on how to conduct effective surveys as part of their training in veterinary epidemiology. We briefed members of the field team with the survey protocols and survey design.

### Sample size

We anticipated a minimum of two hundred respondents for each of the studied districts, however a total of 1466 respondents from all districts who were at least 15 years old and enrolled in ninth grade or higher at educational institutions were included in the study. The sample size was calculated by convenience sampling as the non-probability sampling method rather than probability sampling. Various risk factors for bat-borne zoonoses were evaluated by comparing respondents’ perceptions, attitudes, and practices. The residential backgrounds in this study were known to have differences in their literacy rate, population density, and percentage forest cover with habitat loss [34].

### Survey questionnaire

A questionnaire covering three different sections was prepared and used for data collection. These three sections contained questions regarding each participants demographics, bat-human interactions, and knowledge of bats borne zoonotic disease, primarily rabies [35]. All questions, except for the respondent’s name and level of education, were close ended.

Demographic characteristics that were used included the respondent’s level of education, age, and gender. Awareness of prevention and control of rabies were also evaluated regarding the attitudes and practices of respondents seeking medical attention after a suspected bat bite.

### Statistical analysis

An initial descriptive analysis and cross tabulation of the variables were performed using SPSS 26.0. Bat-borne zoonotic risk factors and different avenues of bat-human interactions were compared in context with the respondents’ residential backgrounds. The respondent’s level of education and their knowledge regarding bat-human interactions were visualized using frequency tables.

The the second step statistical analysis was performed using two multivariate binary logistic regression models (GLMs) in R software. The models were used to evaluate the relationship of residential background of respondent (topographic vs provincial) with three categorical response variables:

- reported suspected bat bite over the lifetime,
- respondents neglect towards managing reported suspected bat bite incidents, and.
- human rabies related deaths in near or extended family member.

**Fig. 2:**
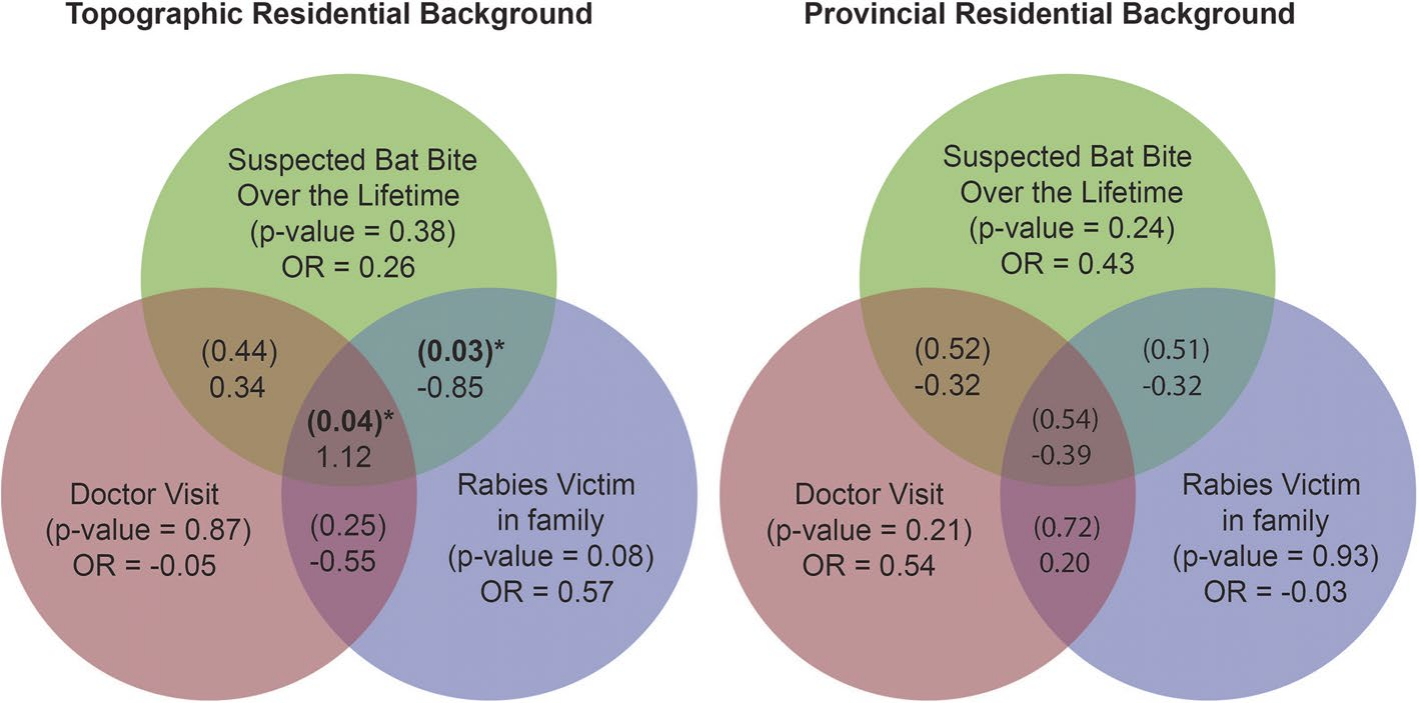
Risk prediction in bat human interactions for two different residential background, A Cross-Sectional Survey on Fruit Bat-Human Interaction in Pakistan; One Health Perspective, 2022

The associations between respondent residential background (topographic vs provincial) were examined using two binary logistic regression models (Fig. 2). Results from the final analysis were expressed in terms of odd ratio and *p*-values with associated 95% confidence intervals We considered these relationships significant at *p* < 0.05 [18]. We used Microsoft 365 (Microsoft®, Seattle, WA, USA) and Microsoft Excel 2019 (Microsoft®, Seattle, WA, USA) for table design, ArcGIS Pro for map display, and Abode Illustrator for Ven diagrams.

## Results

Our study population (*n*  =  1466) had a varied educational and residential background. We compare our results between different residential contexts of respondents to understand which context generated statistical significance describing rabies ecology and epidemiology in Pakistan. The response rate for each question on the questionnaire was 100%.

### Demographic characteristics of respondents

Punjab and Khyber Pakhtunkhwa provinces are comprised of a heterogeneous landscape topography (Fig. 1). Respondents living in the Plain topographic regions were predominantly from Punjab province (99%). Mountain dwellers were unevenly distributed between both the Punjab (64%) and the KPK (36%) province.

Most of the respondents of this survey were of age 18 years or less (70%), residents of Punjab province (79%), predominantly male (64%), of mid-range (Matric, Intermediate, and B.A.) educational level (84%), lived in a mountainous topography (57%), and from primarily a rural background (64%) (Table 1).

**Table 1:** Demographic characterisitic of study respondents. A cross-sectional survey on fruit bat-human interaction in Pakistan; one health perspective, 2022

Variable	Frequency	Percentage (%)
Topographic residential background		
Mountain	834	56.9
Plain	632	43.1
Provincial residential background		
Punjab	1157	78.9
KPK	309	21.1
Education level		
Middle	125	8.5
Matric	487	33.2
Intermediate	462	31.5
B. A	287	19.6
D.V.M	52	3.5
Master	52	3.5
Age		
Age ≤ 18	1015	69.2
Age > 18	451	30.8
Gender		
Men	937	63.9
Women	529	36.1
Background		
Rural	930	63.4
Urban	536	36.6

### Bat-borne disease exposure and residential backgrounds

Overall, 54% of the respondents reported they owned pets or domestic animals. Cross tabulation showed that most of our respondents from mountainous residential background reported keeping pets or animals (61%) in their household, compared to the respondents in the plain regions (44%). Similarly, more respondents had pets in Punjab (56%) compared to pet ownership in the KPK province (44%).

Most of the respondents (75%) reported a general lack of awareness about rabies. It was assumed that a rabies-related death in their close or extended family (29%) was due to their lack of knowledge and failure to seek treatment. 51% of the respondents from a mountainous region were aware of the role of bats in the spread of rabies compared to only 39% respondents in the plain regions (*p* < 0.05, OR 1.69, 95% CI). Most of the respondents from all regions reported seeing evidence of bats near their homes (range 70–75%). A higher percentage of the respondents living in the plain regions (75%) reported seeing evidence of bats near their homes in comparison with mountain dwellers (70%). The difference was found to be statistically significant (*p* < 0.05, OR 0.77, 95% CI).

More respondents from mountainous regions (32%) and KPK province (46%) had noticed that bats left fruit discarded in their gardens compared to those respondents from a plain region (23%) and Punjab province (24%). A minority of respondents (28%) reported fruit discarded by bats in their gardens. Those individuals who saw discarded fruit in their garden also reported a suspected bat bite over their lifetime (32%), twice as high than those who had not seen discarded fruit (16%).

Despite the lack of evidence for the presence of hematophagous bats in Pakistan, 45% of our respondents were concerned that their regional bat populations were “blood-feeding”. Only those respondents with a provincial residential background were found to be statistically significant compared to those respondents with a topographic residential background (*p* < 0.05, OR 1.36, 95% CI) (Table 2).

**Table 2:** Respondents’ responses in relation to their residential backgrounds, a cross-sectional survey on fruit bat-human interaction in Pakistan; one health perspective, 2022

Variables	Topographic residential background				Provincial residential background			
	*p*-value	Odd ratio	Mountainous (*n* = 834)	Plain (*n* = 632)	*p*-value	Odd ratio	Punjab (*n* = 1157)	KPK (*n* = 309)
Presence of pets or any other animal in your house	0.000	1.98 (1.60–2.44)	508 (61%)	278 (44%)	0.001	1.606 (1.24–2.06)	649 (56%)	137 (44%)
Aware bats have role in spread of rabies	0.000	1.668 (1.35–2.06)	427 (51%)	244 (39%)	0.001	0.646 (0.50–0.83)	503 (46%)	168 (54%)
Observed bats in their area	0.028	0.770 (0.60–0.97)	585 (70%)	476 (75%)	0.815	1.034 (0.78–1.37)	839 (73%)	222 (72%)
Knowledge of which bats can feed on blood	0.658	0.954 (0.77–1.17)	373 (45%)	290 (46%)	0.016	1.36 (1.06–1.77)	542 (47%)	121 (39%)
Have a suspected bat bite over lifetime	0.006	1.445 (1.11–1.87)	193 (23%)	109 (17%)	0.491	0.899 (0.66–1.21)	234 (21%)	68 (22%)
Have seen bats discard food (e.g., fruit)	0.000	1.531 (1.21–1.94)	266 (32%)	148 (23%)	0.000	0.375 (0.29–0.49)	274 (24%)	140 (46%)
Aware of rabies vaccine preventable disease	0.277	1.133 (0.905–1.41)	592 (71%)	432 (68%)	0.659	0.940 (0.71–1.24)	805 (70%)	219 (71%)
Knowledge of a rabies awareness campaign in their area	0.400	1.108 (0.87–1.40)	218 (26%)	153 (24%)	0.316	0.865 (0.65–1.15)	286 (25%)	85 (28%)
Aware of clinical signs associated with rabies	0.860	0.981 (0.80–1.21)	363 (45%)	278 (44%)	0.00	1.406 (1.08–1.82)	526 (46%)	115 (37%)
Have visited a doctor after bitten by a bat or a dog	0.000	1.643 (1.33–2.04)	381 (46%)	214 (34%)	0.175	1.196 (0.92–1.55)	480 (42%)	115 (37%)
Rabies in a near or extended family member	0.087	1.221 (0.972–1.54)	260 (31%)	171 (27%)	0.01	0.721 (0.55–0.94)	323 (28%)	108 (35%)

21% of the survey respondents reported a suspected bat bite over their lifetime. A higher proportion of respondents from the mountainous districts (23%) reported suspected bat bite over their lifetime, compared to the plain region respondents (17%). Topographic residential backgrounds (Mountain and Plain) revealed no significant difference in reporting a suspected bat bite over their lifetime (*p*-value = 0.38, OR = 2.6, 95% CI) compared to those respondents from a provincial residential background (Punjab and KPK) (*p*-value = 0.24, OR = 0.43, 95% CI). However, topographic residential backgrounds did reveal a significant difference in reporting a suspected bat bite over the lifetime with a rabies related death in near or extend family member (*p* = 0.03, OR = −0.85., 95% CI) than provincial residential background (*p*-value = 0.51, OR = 0.3, 95% CI) (Fig. 2).

Overall, 74% of the respondents from all backgrounds (Punjab, KPK, Mountainous, Plain) were aware of rabies as a vaccine preventable disease. Slightly more than half of the respondents (55%) lacked knowledge of the transmission of rabies from bats to humans. Less than half (44%) of our respondents were knowledgeable of the clinical signs of rabies. The practice of seeking medical care following a suspected bat bite for wound management was observed to be 41%.

### Risk predictor for contracting bat rabies in different residential backgrounds

**Fig. 3:**
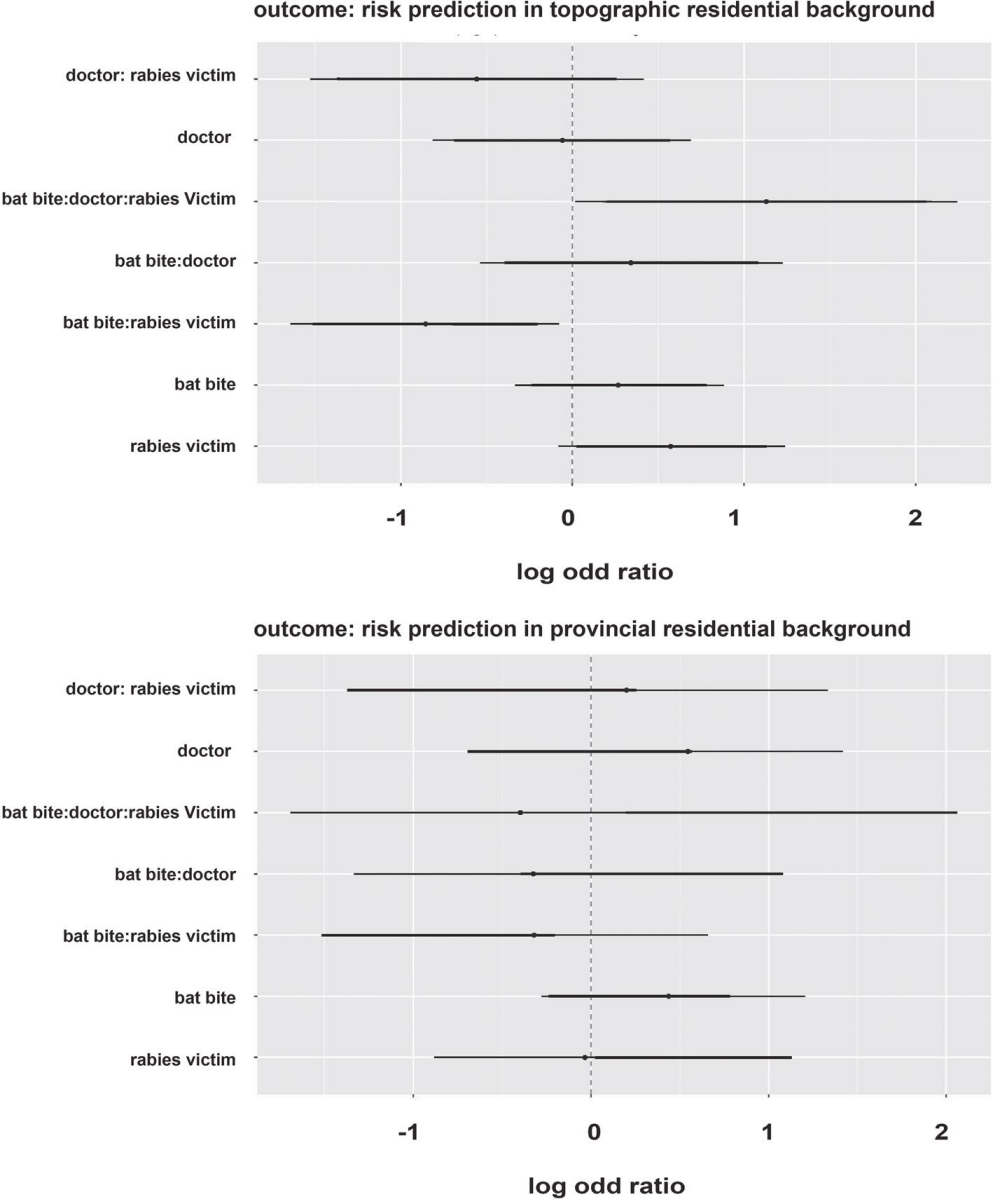
Logistic regression model cofficients plots for risk prediction in bat human interacting in pakistan, A Cross-Sectional Survey on Fruit Bat-Human Interaction in Pakistan; One Health Perspective, 2022

Our multivariate binary logistic regression models highlighted topographical residential background had more association in reporting suspected bat bite over the lifetime and rabies related human deaths in a near or extended family member. Few respondents from all backgrounds were aware of thet clinical signs of rabies (x̄ = 42%) and the availability of a rabies awareness campaign (x̄ = 25%) in their area (*p* < 0.05, OR = 1.1, 95% CI) (Table and human rabies related deaths in combination with negligence in visiting the doctor after reported suspected bat bite in comparison with resident of provincial residential backgrounds (Punjab & KPK) (Figs. 2 and 3).

## Discussion

Our cross-sectional study assessed the knowledge, attitudes and practices in a select region of Pakistan regarding fruit eating bats and their associated pathogens and explore avenues of bat-human interactions that could lead to pathogen spillover. We asked questions regarding the presence of bats in their surroundings and suspected bat bite over the lifetime of the respondent. This study also asks a question regarding the presence of suspected fruits discarded by bats as an indicator of potential bat-human interaction*.* The results of the study will increase our ability to identify possible hotspots of bat-human interaction with potential for pathogen spillover in Pakistan. People living in the mountainous areas of Pakistan have more frequent interactions with bats, have a higher reported incidence of a suspected bat bite over their lifetime, and a family member with a suspected rabies related mortality. However, this preliminary cross-sectional study does not confirm an association between exposure (i.e. scratches in particular) and risk of rabies infection due to lack of serological evidence of lyssaviruses in bats in Pakistan [33]. The low levels of awareness and understanding about the beneficial role of bats in the ecosystem, coupled with suspected associated risk of bats’ borne zoonoses has led to mass persecution of bats in many parts of world including the Indian subcontinent [36–38].

The Indian flying fox is a common fruit bat species in the Indian subcontinent and is capable of flying long distances for daily foraging [39]. These bats have an important role as pollinators and long-distance seed dispersers in tropical ecosystems by chewing fruit, obtaining juice and pulp, then spitting seeds [40]. These fruit bats are facing widespread persecution despite its critical role in providing valuable ecological services [8, 9, 41]. Unfortunately, farmers and governments often view this species as vermin, leading to inhumane treatment [40, 42]. The Covid-19 pandemic has exacerbated this issue globally [37].

Studies showed zoonotic virus transmission can occur through food contaminated by bat viruses or eating bats as bushmeat [43]. Climate change drives conflict between bats and humans via habitat degradation and food resource fluctuations [44]. Our unpublished work also found that climate change induced extreme heat events has resulted in die-offs of Indian flying foxes in Pakistan [45] and India [46] and has potentially lead to change in their geographic niche. Extreme heat is not only a conversation challenge but it can results in pathogen spillover [47].

Over 70% of the respondents from all backgrounds (provincial and topographic) have reported the presence of bats in their areas. This finding is consistent with study in Bangladesh [48] and Pakistan [9]. On an average, 30% of our respondents reported observing the presence of fruits discarded by bats as an indicator of bat-human interaction. This finding is similar to the findings of previously reported study where 31% respondents observed bats in their courtyard or compound [48].

Most of the study respondents in both regions (topographical and provincial) were aware of the mode of rabies virus transmission. All respondents were also aware of the bats’ role in rabies dispersal. This finding is consistent with previous studies conducted in South Asia [49]. This study also noticed higher incidents of reported suspected bat bite over the lifetime in mountainous residential communities and more rabies related human deaths in KPK province. This finding can be explained in several ways. There is a reported relationship of intensive deforestation with an increase in bat bite incidents and lyssavirus related human deaths in the mountainous areas (99% of our surveyed KPK province residents had a mountainous residential background).

According to the World Health Organization, any contact with a bat is a category III exposure requiring post exposure prophylaxis (PEP) [50]. A rabid bite victim requires immediate PEP for survival. The poor infrastructure in rural areas of KPK province due to mountainous areas, can limit peoples’ movement to seek and access health care facilities after an animal bite. Consequently, seeking health care facilities immediately after animal bites was much lower in mountainous districts in this survey.

The practice of visiting health care facilities and satisfaction with the available facilities was lower than many previous studies around the globe [51–53]. We can indirectly improve respondents’ practice of visiting health care facilities and reporting suspected animal bites, by increasing awareness regarding cross-species pathogen transmission [54]. A better understanding of how bats spread pathogens and what to do if bitten is crucial to preventing rabies and other infectious diseases [55].

Previous studies conducted in Pakistan found that majority of the study respondents negatively perceived bats as sign of bad omen [9, 26]. 46% of our study respondent were concerned that local bats could feed on blood despite there is no reported presence of hematophagous bats species in Pakistan. This result in our study is inconsistent with the findings of a previous study which reported just 3.1% respondents had such belief about bats in Pakistan [26]. We assume that the most of the respondents’ knowledge of a hematophagous feeding strategy in bats is a negative perception due to the absence of hematophagous bat species in Pakistan [9]. It would be interesting to investigate the ways did the respondents learn about bats and how they developed this negative perception of blood feeding among the local bat population. In general, people lack direct experience with wildlife and form their risk perception primarily based on media reports. By framing news differently, the media may significantly impact public perception of risk, thereby promoting or discouraging public tolerances [36–38].

Approximately 28% of our study respondents reported bats discarded fruits in gardens. This finding is a reflection of what Pakistani orchard farmers (62%) reported damage attributed to fruit bats in their fields [9]. Additionally, our study shows that 32% of those who saw bats’ discarded fruits in their garden had twice the chance of getting bat bites compared with those who did not. This result highlights the importance of a recent study in Pakistan, where 71% of respondents considered bat contaminated fruits as a potential source of pathogen transmission [9]. Fruit contamination from bat urine and saliva has previously been linked to public health concerns [56]. In our study approx. 50% of people think that bats are the source of spreading rabies. This finding can be related to a recent study in Pakistan [9] and Argentina [57], where approximately 42% of the local people and farmers believed that bats transmit pathogens.

Chiropteran rabies is frequently neglected in Pakistan due to absence of bat lyssavirus related human mortalities. However, the presence of lyssavirus and its variants in bats species in regional countries can contribute to rabies outbreaks. Mountainous regions in the Khyber Pakhtunkhwa province have a comparatively high biodiversity of insectivorous (Molossidae and Vespertilionidae) and frugivorous bats (Pteropodidae) which are recognized as a lyssavirus reservoir [58]. We therefore suggest that clinicians should ask suspected rabies patients about contact with bats. Our findings underscore the necessity of serological surveillance to determine the presence and frequency of bat lyssaviruses and other bat-borne zoonotic pathogens in Pakistan.

This study has some limitation, including age group of respondents (>16 years of age) and no cross verification of bat bite incidents. Additionally, the data used in this analysis was not collected primarily for the purpose of studying bat-human interaction. An open-ended survey dedicated to studying bat-human interactions would provide a much more in-depth understanding of bat–human interactions [48].

## Conclusions

This survey highlights close interactions of fruit eating bats with humans. These bats are a recognized pathogen carrier and reservoir of zoonotic pathogens including rabies in the Indian subcontinent. As no study has been conducted to determine the role of fruit eating bats in spreading lyssavirus in Pakistan, we conclude based on the results of our survey that a bat component should not be ignored in any national rabies surveillance. The inclusion of bats, considering companion animals as a significant predator of live and deceased bats, will provide valuable insight into the rabies cycle and aid in saving life of humans and animals. Topographic (mountains and plains) residential background is significantly associated with the rate of bat-human interaction as compared to provincial (Punjab and KPK) residential background and could be used to identify potential hotspots of bat-borne pathogen.

## Data Availability

The datasets generated and analyzed during the current study are available in the Dryad repository; Ahmed, Touseef (2021), A Cross-Sectional Survey on Bat Human Interaction in Pakistan; One Health Perspective https://doi.org/10.5061/dryad.8sf7m0chp.
